# Regional Risk Evaluation of Flood Disasters for the Trunk-Highway in Shaanxi, China

**DOI:** 10.3390/ijerph121113861

**Published:** 2015-10-29

**Authors:** Hong-Liang Qi, Wei-Ping Tian, Jia-Chun Li

**Affiliations:** Key Laboratory for Special Area Highway Engineering of Ministry of Education, Chang’an University, Xi’An 710064, China; E-Mails: fz02@gl.chd.edu.cn (W.-P.T.); chdlijiachun@126.com (J.-C.L.)

**Keywords:** highway engineering, flood disaster, risk evaluation, trunk-highway, GIS

## Abstract

Due to the complicated environment there are various types of highway disasters in Shaanxi Province (China). The damages caused are severe, losses are heavy, and have rapidly increased over the years, especially those caused by flood disasters along the rivers in mountainous areas. Therefore, research on risk evaluations, which play important roles in the prevention and mitigation of highway disasters are very important. An evaluation model was established based on the superposition theory of regional influencing factors to highway flood disasters. Based on the formation mechanism and influencing factors of highway flood disasters, the main influencing factors were selected. These factors include rainstorms, terrain slopes, soil types, vegetation coverage and regional river density, which are based on evaluation indexes from climate conditions and underlying surface of the basin. A regional risk evaluation of highway flood disasters in Shaanxi was established using GIS. The risk index was divided into five levels using statistical methods, in accordance with the regional characteristics of highway flood disasters. Considering the difference in upfront investments, road grade, etc, between expressways and trunk-highways in China, a regional risk evaluation of trunk-highway flood disasters was completed. The evaluation results indicate that the risk evaluation is consistent with the actual situation.

## 1. Introduction

**Figure 1:**
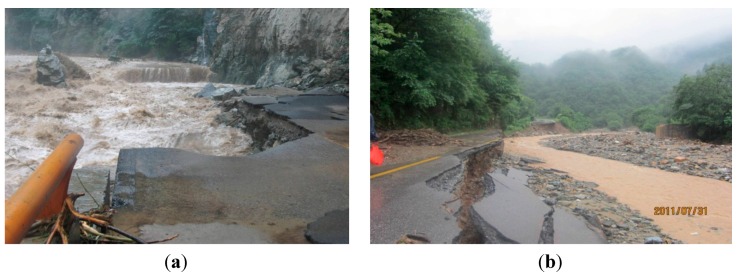
Flood damages to highways. (**a**) Whole-width damage; (**b**) Half-width damage.

Due to the complicated environmental conditions, losses caused by different types of highway disasters in Shaanxi Province are very heavy. Highway flood disasters are the top disaster for highways, which has increased rapidly in the past years (Figure 1). They severely restrict the highway construction and economic development of Shaanxi Province. Therefore, research on risk evaluation for highway flood disaster is of great importance. Those achievements will play important roles in guidance of the prevention and mitigation of regional highway disasters.

Researches on risk evaluation of disasters have been conducted in recent years, producing numerous results [1,2,3,4,5]. The regional risk of new coastal flood hazards along the New England shoreline was studied by Caufield and Hillier [6]. In this study, new detailed coastal flood hazard analysis and mapping of the community areas along the shoreline were provided. With the development of computer technology, Geographic Information System (GIS) technology has been widely applied in the regional disaster evaluation field [7,8,9,10,11], and has been very useful. GIS technology provides a reference for regional road-network planning, disaster prevention and reduction. In the areas of risk evaluation of highway natural disasters, systematic research regarding the theory and methods of vulnerability and risk evaluation of highway geological disasters has been conducted by Qi [12]. Meanwhile, systematic research on the technology of highway flood damage identification has been conducted by Ma [13]. In addition, a study on the evaluation and prediction of regional highway slope disasters based on GIS has been conducted by Wang [14]. A research on database creation and risk evaluation of highway geological disasters in Southern Shaanxi with GIS was reported by Yang [15].

In summary, most previous studies were on single point highway natural disasters. Research on regional risk evaluation of highway flood disasters based on GIS was rarely reported. This paper therefore focuses on the regional risk evaluation of highway flood disasters in Shaanxi, providing a reference and guidance for regional trunk-highway flood disaster prevention.

Firstly, highway flood disasters can be variously classified per different focuses. Therefore, highway flood disasters mainly caused by rainstorm are selected as the research subject in this paper. Secondly, a model of regional risk evaluation is established based on the superposition theory of regional influencing factors. Risk evaluation highway flood disaster factors are examined from two aspects, which include climate and the underlying surface of the basin. Thirdly, evaluation index grading and their weight are determined by the model. Finally, a regional risk evaluation and risk distribution of trunk-highway flood disasters in Shaanxi Province are carried out by GIS.

## 2. Highway Flood Disasters

According to a literature review, there is no generally accepted classification method for highway natural disasters. Highway natural disasters were classified into four types in the literature [16], which include geological disasters, meteorological disasters, environmental pollution disasters and soil disasters. In [17], highway flood disasters are considered to be a series of damage processes and phenomena affecting a highway under the combined effects of climate, hydrology, geology environmental factors and human activities, which was a general designation of various disasters suffered by highways. In conclusion, highway disasters were classified into four types by field surveys: collapses and landslides, mudslides, highway flood damages and frothing. However, there are many troubles in practical application due to various reasons, such as: (1) the classification of highway disaster type is not unified because of the differences between research perspectives, emphasis, industry characteristics, *etc.*; (2) types of highway natural disasters classification are inconsistent with other disciplines; (3) many researchers have focused on several common disasters like landslides, mudslides, floods *etc*.; (4) researches on disaster management from a macroeconomic perspective are few.

In this paper, according to their occurrence mechanism and main influencing factors, highway natural disasters were classified into two types: geological disasters (also called meteorological-geological disasters) and meteorological disasters. Highway geological disasters are those geological disasters caused by the deterioration of environmental conditions due to geological, rainfall and other meteorological factors or human activities. Geological conditions are the fundamental causes. Meteorological factors and human activities can induce and accelerate the disaster occurrence. According to the formation mechanism, typical geological disasters include earthquakes, collapses, landslides, mudslides and subsidence, *etc.* Highway meteorological disasters, including damages to highways and their subsidiary facilities, or decreased functions and economic losses, are caused by atmospheric condition changes. Typical highway meteorological disasters are fog disasters, frozen disasters, snow disasters and highway flood disasters as described by their formation mechanism. Damages caused by highway flood disasters, including roadbed damage, small bridge and culvert damage, slope erosion, flooding and soaking, are the most serious and common ones in Shaanxi. Among the many options available, highway flood disasters caused mainly by rainstorms were selected as the research subject of this paper.

## 3. Model of Regional Risk Evaluation

*R* was set to be the risk level of regional highway flood disaster. It was used to characterize the degree of risk that a highway may suffer under certain climatic conditions, like rainstorms, topography, geological conditions and other basin underlying surface conditions.

Based on the superposition theory of regional influencing factors, an equation was established as follows:

(1) $$R=\sum_{i=1}^{n}F_{i}\cdot W_{i}$$

where: *R*: risk level of regional highway flood disaster; *F_i_*: the grades of risk evaluation indices; *W_i_*: the weights of risk evaluation indices; *n:* the number of risk evaluation indices. Evaluation indices, and their grades, as well as their weights need to be established first based on the above model.

### 3.1. Grades of Risk Evaluation Indices

#### 3.1.1. Main Risk Factors

The mechanisms of highway flood disasters are very complex, especially for those along rivers located in mountainous areas. There are many influencing factors, while the interrelationship among them can be very complicated. Increases in the regional runoff, especially the river runoff, can effectively increase the velocity, the sediment carrying capacity and subsequently the scouring capability of flows. This leads to subgrade collapses, which are a serious highway flood disaster. Therefore, river runoff directly impacts the regional risk of highway flood disasters. The main influencing factors of river runoff are also those most affecting the risk of highway flood disasters.

There are many natural influencing factors according to the runoff formation conditions. These can be divided into climatic factors, and underlying surface factors of the basin and human activities. Rainfall is the major climatic factor that affects runoff. The form, amount, process and spatial distribution of rainfall in the basin have a direct effect on the runoff. The amount of river runoff depends on the amount of rainfall, that is, the river runoff is correlated with rainfall.

The relationship between the formation of runoff and underlying surface factors of the basin is very close. Underlying surface factors are mainly influenced by the regional topography, geological conditions, vegetation characteristics and other conditions. Regional topography, slope for example, is a typical factor of the time of runoff confluence. Regional geological conditions, especially the types of rock and soil, have significant impacts on the infiltration capacity of water. Vegetation coverage affects the loss of rainfall caused by the retention of root, stem and leaf of vegetation.

Human activities affecting runoff change the underlying surface conditions, which directly or indirectly affects the amount and process of runoff. Different combinations of all the above environmental factors lead to different types of runoff.

#### 3.1.2. Indices of Regional Risk Evaluation

Based on the formation mechanism and influencing factors of risk evaluation on highway flood disasters, rainstorm, terrain slope, soil types, vegetation coverage ratio and regional river density were selected as the main indices of regional risk evaluation of highway flood disaster in Shaanxi, as shown in Table 1.

**Table 1:** Indices of regional risk evaluation.

Main Influencing Factor	Evaluation Index	Characteristic Index
climate conditions	rainstorm	average days of rainstorm per year
underlying surface of the basin	soil types	regional soil types
	vegetation coverage	regional vegetation coverage ratio
	terrain slope	regional average slope
	river	regional river density

#### 3.1.3. Grades of Risk Evaluation Indices

Grades of indices are used to characterize different values when a same index targets different levels. Different influencing levels of an index can be determined by grading and scaling it. The index grading is the key point. Considering the actual conditions of highway engineering, and related researches [18,19,20,21], the grading and scaling of indices of regional risk evaluations of highway flood disasters in Shaanxi were established as shown in Table 2.

**Table 2:** Grades of risk evaluation indices.

Evaluation Index	Grade				
	Slight	Light	Medium	Severe	Extremely Severe
Average days of rainstorm per Year (d)	˂0.7	0.7–1.2	1.2–2.2	2.2–3.7	>3.7
Average slope (°)	≤3	3–10	10–15	15–25	>25
Soil types	Loess and Silt	Sandy soil	Cohesive Soil	Gravel soil and soft rock	Hard Rock
Vegetation coverage ratio (%)	>80%	50%–80%	20%–50%	5%–20%	0%–5%
river density (m/km^2^)	0–500	500–1500	1500–2500	2500–3500	>3500
F_i_	1	3	5	7	10

### 3.2. Weight of Indices

The weight of every index is defined in Table 3. The value of every coefficient was defined by the scores of ten recognized specialists in the highway design and construction fields.

**Table 3:** Weight of indices.

Index	Average Days of Rainstorm Per Year	Average Slope	Soil Types	Vegetation Coverage Ratio	River Density	Total
W_i_	0.3	0.2	0.2	0.2	0.1	1

## 4. Regional Risk Evaluation

The regional data of rainstorm days in Shaanxi was obtained from the *Shaanxi Meteorological Disaster Atlas (1961–2006)*. The average slope data of ground calculations was obtained from a 1:25 million Digital Elevation Model (DEM) of Shaanxi. That of the soil types in Shaanxi came from the *Engineering Geological Map of Shaanxi*. The regional vegetation coverage ratio data was obtained from the *Vegetation Map of Shaanxi*. Regional river density data came from the *River System Map of Shaanxi*. The characteristic indices were analyzed by GIS and the grid size was 100 m×100 m. Based on Table 2 and Table 3, the highway flood disaster risk in Shaanxi Province was established by GIS using Equation (1). The results and the regularity of distributions are shown below.

**Figure 2:**
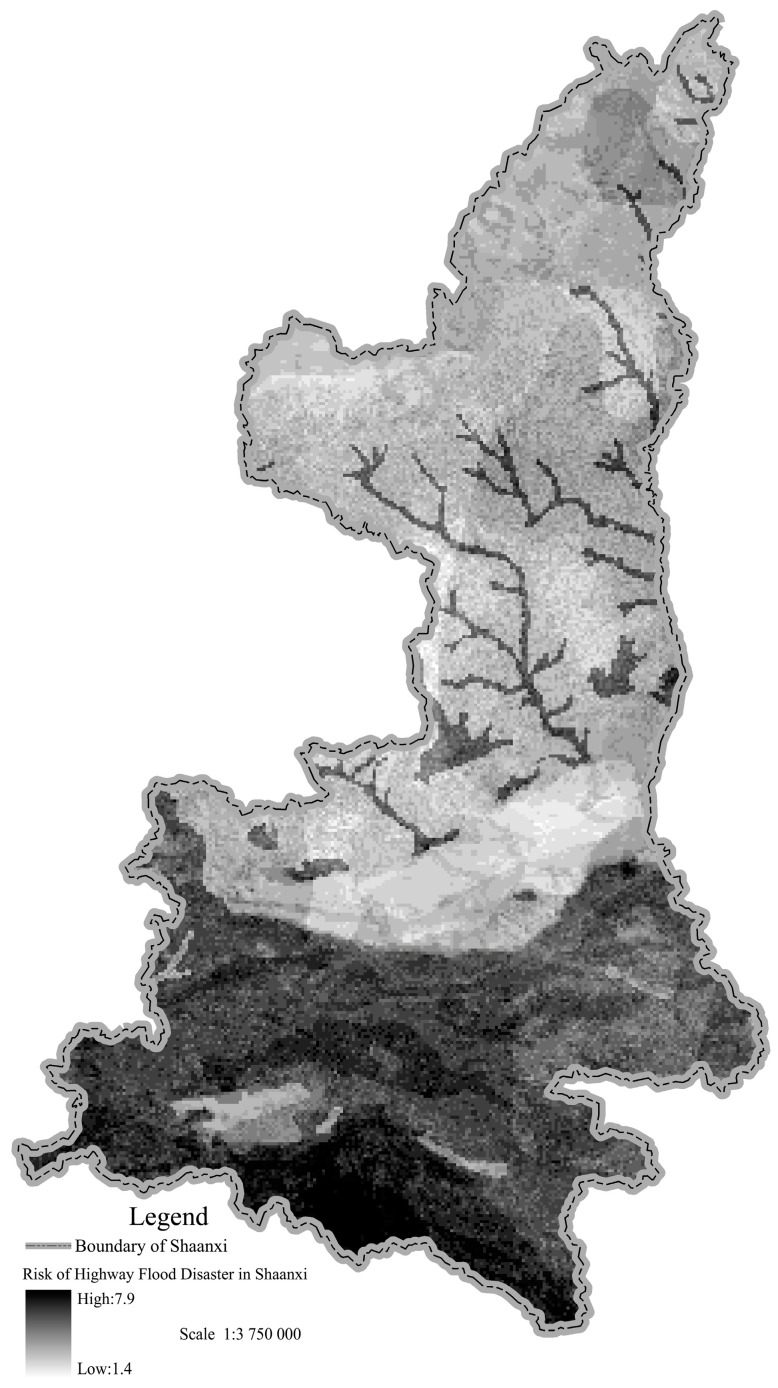
Distribution of highway flood disaster risk in Shaanxi Province.

In Figure 2, there are obvious regional characteristics in the distribution of the highway flood disaster risk indexes. The maximum value is in Qingling-Bashan mountainous areas, while relatively low values are in the Guanzhong plain, Hanzhong basin and Maowusu desert at northwest Yulin. Most parts of the loess plateau in northern Shaanxi are medium risk. The reason is that the average days of rainstorm per year in Qingling-Bashan mountain area are the largest in Shaanxi Province (the most part is above 3.2 days, while the number in Zhenba it can even be 4.9 days). Meanwhile, the slopes and the river density in those areas are larger. The ground surface is covered by rocks, while the loss of rainfall is less. However, the average days of rainstorm per year in the Guanzhong plain and Maowusu desert in northwest of Yulin is less than 0.7 days, while the slopes in the area are smaller. Meanwhile, the ground surface is covered by Quaternary loess, and the loss of rainfall is higher.

## 5. Regional Risk Evaluation of Trunk-Highway

For all kinds of highways, the upfront investment in expressways is so large that the corresponding disaster prevention and measurement system is complete. Natural disasters have little effect little on them. Trunk-highways in China were constructed in earlier years. Road grades were lower due to technical and economic limitations. This can be improved by a suitable disaster prevention and measurement system. Threats of natural disasters are greater, and economic losses is higher, so the regional risk evaluations of trunk-highway flood disasters are more urgent.

In order to take appropriate preventive measures more easily, *R* needs to be graded. Based on the normal features and the regional characteristics of highway flood disasters in Shaanxi, *R* was divided into five levels as shown in Table 4 by using the Natural Breaks (Jenks) method provided in GIS.

**Table 4:** Risk level of highway flood disaster in Shaanxi province.

Risk Level	Slight (I)	Light (II)	Medium (III)	Severe (IV)	Extremely Severe (V)
Risk index	<2.4	2.4–3.4	3.4–4.8	4.8–5.8	>5.8

**Figure 3:**
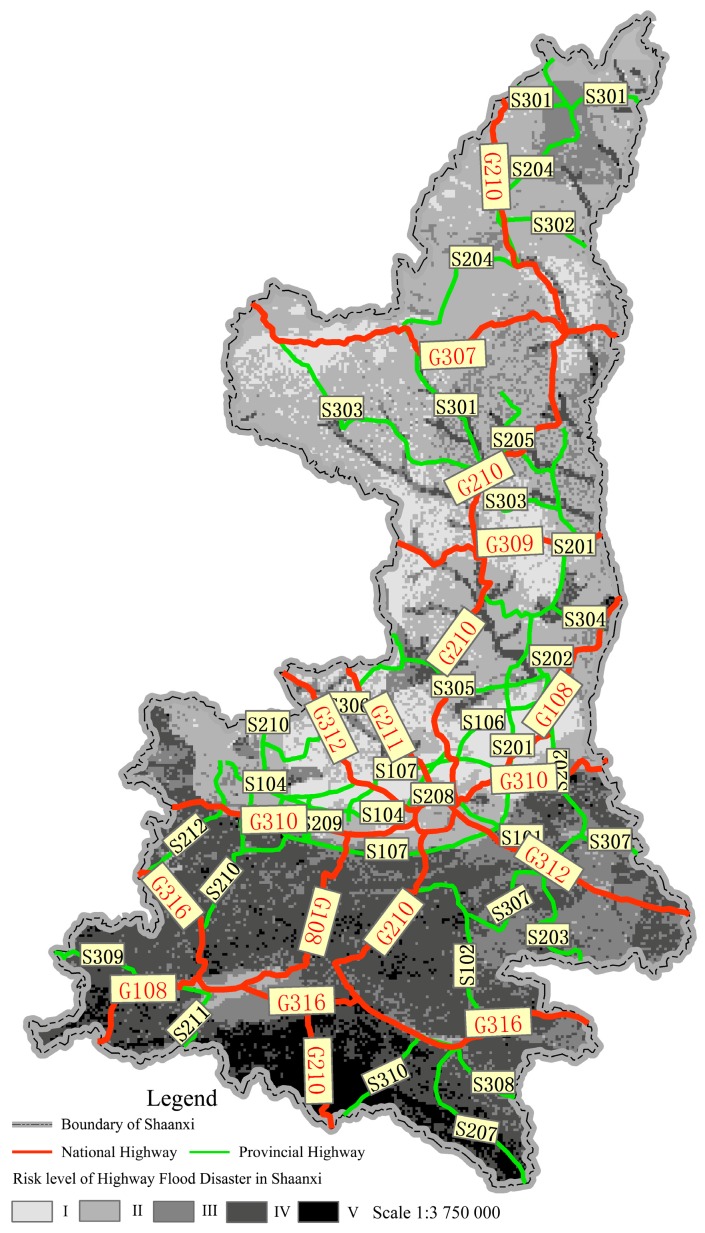
Distribution of trunk-highway flood disaster risks in Shaanxi Province.

There are eight national highways and twenty-four provincial highways in Shaanxi. The distribution of trunk-highway flood disaster risks in Shaanxi is shown in Figure 3.

G108 is one of the national highways. It is an important route from Shaanxi Province to the southwest of Sichuan. From k1372+500 to k1461+090, G108 was constructed along the HeiHe River in the northern foothills of the Qinling Mountains. The risk levels of highway flood disaster in this area cross severe (IV) and extremely severe (V) according to the study.

From 5 P.M. 28 July 2011 to 8 P.M. 29 July 2011, heavy rains fell in Qinling Mountains. The rainfall rose to 200 mm, which caused severe damages to G108. Those caused by floods were extremely heavy. There were four sections of whole-width damage to the subgrade (370 m in length), five sections of half-width damage to the subgrade (235 m in length), 24 sections with damage to lower retaining walls (2.2 × 10^4^ m^3^ in volume) and 76 sections of damage to the pavement (6000 m^2^ in surface). Traffic was disrupted for one whole month and the economic losses were at least 38 million RMB. The regional risk evaluation results of this study are thus consistent with the actual situation.

## 6. Conclusions

A systematic and thorough research on regional risk evaluation of highway disasters caused by rainstorms was conducted in this paper. A regional risk evaluation model was established. Factors and indices that affect the most with their grading and weight were identified. With detailed information, a risk evaluation figure for the trunk-highways of Shaanxi Province was carried out by GIS. The conclusions are as follows:

1. An evaluation model was established according to the superposition theory of regional influencing factors to highway flood disasters. R was used to characterize the degree of regional highway flood disaster risk that highways may suffer under different environmental conditions.
2. Climate factors, underlying surface factors of the basin and human activities were considered as the main natural influencing factors. Based on the formation mechanism and influencing factors of risk evaluation on highway flood disasters, rainstorms, terrain slopes, soil types, vegetation coverage ratio and regional river density were selected as the main indices of regional risk evaluation of highway flood disaster in Shaanxi.
3. Grades of evaluation indices and their weights were prompted according to the evaluation model. A regional risk evaluation of highway flood disaster in Shaanxi Province was carried out by GIS, and it was divided into five grades: slight (I), light (II), medium (III), severe (IV) and extremely severe (V).
4. A distribution of trunk-highway flood disaster risks in Shaanxi was put forward. The risk evaluation results are consistent with actual situations.

This research will provide guidelines for highway planning and construction in the future. Risks can be easily identified. More reasonable effective measures can be considered accordingly. The achievements will have more positive effects through wider promotion and application.
